# SIRT4 controls the balance between lipid synthesis and catabolism by repressing malonyl-CoA decarboxylase

**DOI:** 10.1186/1753-6561-6-S3-P30

**Published:** 2012-06-01

**Authors:** Gaëlle Laurent, Natalie J German, Asish K Saha, Vincent CJ de Boer, Frank Fischer, Gina Boanca, Noah Dephoure, Bhavapriya Vaitheesvaran, Michael Davies, Steven P Gygi, Deborah M Muoio, Irwin J Kurland, Clemens Steegborn, Neil B Ruderman, Marcia C Haigis

**Affiliations:** 1 Department of Cell Biology, The Paul F. Glenn Labs for the Biological Mechanisms of Aging, Harvard Medical School, Boston, MA 02115, USA; 2 Diabetes Research Unit, Section of Endocrinology, Department of Medicine, Boston University Medical Center, Boston, MA 02118, USA; 3 Current address: Laboratory Genetic Metabolic Diseases, Academic Medical Center, Amsterdam, 1105AZ, The Netherlands; 4 Department of Biochemistry, University of Bayreuth, 95447 Bayreuth, Germany; 5 Department of Cell Biology, Harvard University Medical School, Boston, MA 02115, USA; 6 Department of Medicine, Diabetes Center, Stable Isotope and Metabolomics Core Facility, Albert Einstein College of Medicine, Bronx, New York 10461, USA; 7 Departments of Medicine and Pharmacology & Cancer Biology, Sarah W. Stedman Nutrition and Metabolism Center, Duke University Medical Center, Durham, NC27710, USA

## 

Lipid metabolism is highly controlled by the nutritional state of the organism. In this study, we identify the mitochondrial sirtuin, SIRT4, as a critical regulator of lipid homeostasis. We find that SIRT4 represses fatty acid oxidation while promoting lipid anabolism. Mechanistically, SIRT4 regulates this balance by inhibiting malonyl-CoA decarboxylase (MCD), an enzyme that produces acetyl-CoA from malonyl-CoA, a precursor for lipogenesis that also inhibits mitochondrial fat oxidation. We find that SIRT4 is active in nutrient-rich conditions, such as in the fed state. As a consequence, SIRT4 null mice display reduced levels of malonyl-CoA in skeletal muscle and white adipose tissue in the fed state and fail to further lower malonyl-CoA levels during fasting. SIRT4 null mice possess a catabolic signature of lipid metabolism and demonstrate decreased de novo lipogenesis. These studies highlight SIRT4 as a novel regulator of MCD activity and malonyl-CoA levels, providing new insight into the regulation of lipid homeostasis.

